# Sensory and Quasi-Sensory Experiences of the Deceased in Bereavement: An Interdisciplinary and Integrative Review

**DOI:** 10.1093/schbul/sbaa113

**Published:** 2020-10-25

**Authors:** Karina Stengaard Kamp, Edith Maria Steffen, Ben Alderson-Day, Paul Allen, Anne Austad, Jacqueline Hayes, Frank Larøi, Matthew Ratcliffe, Pablo Sabucedo

**Affiliations:** 1 Department of Psychology and Behavioural Science, Aarhus University, Aarhus, Denmark; 2 Department of Psychology, University of Roehampton, London, UK; 3 Department of Psychology, Durham University, Durham, UK; 4 Department of Psychosis Studies, Institute of Psychiatry, Psychology and Neuroscience, Kings College London, London, UK; 5 Faculty of Theology, Diaconia and Leadership Studies, VID Specialized University, Oslo, Norway; 6 Department of Biological and Medical Psychology, University of Bergen, Bergen, Norway; 7 Psychology and Neuroscience of Cognition Research Unit, University of Liège, Liège, Belgium; 8 Norwegian Centre of Excellence for Mental Disorders Research, University of Oslo, Oslo, Norway; 9 Department of Philosophy, University of York, York, UK

**Keywords:** auditory-verbal hallucination, population, persistent complex bereavement disorder, sense of presence

## Abstract

Bereaved people often report having sensory and quasi-sensory experiences of the deceased (SED), and there is an ongoing debate over whether SED are associated with pathology, such as grief complications. Research into these experiences has been conducted in various disciplines, including psychiatry, psychology, and anthropology, without much crossover. This review brings these areas of research together, drawing on the expertise of an interdisciplinary working group formed as part of the International Consortium for Hallucination Research (ICHR). It examines existing evidence on the phenomenology, associated factors, and impact of SED, including the role of culture, and discusses the main theories on SED and how these phenomena compare with unusual experiences in other contexts. The review concludes that the vast majority of these experiences are benign and that they should be considered in light of their biographical, relational, and sociocultural contexts.

## Introduction

Hallucinations and other unusual sensory experiences are often associated with a psychiatric disorder. However, such experiences also occur in nonclinical populations and in response to specific life events. These include bereavement, where a range of sensory experiences is reported as well as quasi-sensory “feelings” or “nonspecific awareness of presence.” ^1–3^ Prevalence estimates of having at least one of these experiences range from 47% to 82% across several studies.^1,2,4–7^

Although these phenomena are sometimes referred to as “hallucinations,” ^2,4,5,8^ research on this topic uses various terms (see table 1), often reflecting differing theoretical assumptions and encompassing a wider range of phenomena than most uses of the term “hallucination” (the latter often being defined as perceptual experiences that arise in the absence of appropriate stimuli).^9^^(p68),^^10^^(p242)^For instance, a sign, message, or dream visitation might also be said to involve a perception-like experience of receiving a communication from the deceased, as well as a sense of the deceased’s presence.^11^ However, as many of the relevant phenomena are under-theorized and poorly understood, there is no consensus concerning where boundaries should be drawn. Here, the term “sensory and quasi-sensory experiences of the deceased” (SED) is adopted in preference to “hallucinations,” as a more neutral and inclusive term.

**Table 1.** Terms Used to Denote Sensory and Quasi-Sensory Experiences of the Deceased (SED)

Terms	Conceptualizations
After-death communications	“[R]eported encounters with a deceased loved one”.^12^ Bereaved individuals experience what is believed to be actual spiritual contact with a deceased loved one.^13^
After-death spiritual experiences	“[A] postmortem contact with a spirit that feels separate from the living person” (as part of continuing bonds.)^14^
Bereavement hallucinations	“[E]xperiences of seeing, hearing, or sensing the presence of the deceased”.^5^
Continuation of bond initiated by the deceased	“Feel, hear, or see the deceased after the death”.^15^
Experiences of continued presence	Hearing the voice or sounds indicating the activity of the deceased, experiencing an image, feeling the touch of the deceased or smelling them, and feelings of presence unspecified by any of the senses.^3^
Experiences of presence	“[W]hen the bereaved perceive (via hearing, seeing, touching, smelling) or feel (the presence of) the deceased person”.^16^
Extraordinary experiences of the bereaved	“[E]xperiences that occur at the time of, or after the death of someone known to an experient and is assumed by that experient to signify contact or communication with the deceased”.^17^
Ghost illness	A culture-bound syndrome among American Indians; spirits or ghosts linked to events, accidents, or illness.^18^
Grief hallucinations	Denoting a variety of psychic and psychopathological phenomena and may be “true” hallucinations and not pseudohallucinations.^19^ A benign form of coping with bereavement, a type of defense mechanism designed to protect the ego from adverse effects.^20^
Hallucinations and illusions	A type of externalized continuing bond.^21^ Hallucinations of the dead.^22^
Hallucinatory experiences during mourning	Part of the mourning process among Hopi Indian women.^23^
Idiophany	Personal sensory experiences among people without mental illness.^24^
Idionecrophanies	Private appearances and perceived contact with the dead.^25^
Ideonecrophic experience	“[T]he experience of contact from or communication with a deceased being”.^26^
Perceived presence of deceased loved ones	Psychological phenomena that are a natural and generally healthy component of grieving.^1^
Post-bereavement hallucinations	Visual, auditory, or tactile experiences of the deceased, conversation with the deceased, feeling of his or her presence.^2,4,6^
Post-death contact	Mystical or spiritual experience or unmistakable encounter.^27^ “[W]hen a living individual feels that a person who is deceased is reaching out to connect with the living”.^28^
Post-death encounters	Seeing, hearing, or feeling the presence of the deceased; having met with or felt the presence of someone close to them who had died.^29^ Includes sensory experiences (hearing, seeing, being touched by, or smelling something directly related to the deceased) and sense of presence experiences when the loved one feels as though they are in the immediate area for a limited amount of time.^30^
Presence of the dead	Post-bereavement experiences providing ongoing relationship.^31^
Sense of presence/sense of presence experiences/sense of presence of the deceased	A “very vivid internal experience,” “vivid illusions.” ^32^ A way of maintaining the continuing bond.^33^ Expression of the continuing bond/ongoing attachment.^34^ “[N]onmaterial quasisensory subjective but (experienced as) veridical feeling of presence of the deceased”.^11^ Part of religious and cultural practices in bereavement.^35^ The experience of feeling the presence of a deceased person.^36^ “[H]aving a ‘feeling’ that the deceased is present or experiencing them in a sensory modality”.^37^ An anomalous event in bereavement involving “spontaneous sensory phenomena which [experiencers] may interpret as interaction and/or communication with significant people who have died”.^38^
Sensing experiences	Experiencing the presence of the dead: may be auditory, visual, or tactile or simply perceived as an unspecified presence.^39^
Sensory-perceptual experiences of bereaved individuals	“Special experiences” in which an “overwhelming intuitive or sensory presence of a deceased loved one was felt by the bereaved person”.^7^
Sightings of the deceased	“[P]erception of the presence of the deceased through one or more of the five senses”, a type of after-death communication.^40^
Spiritual connections with the deceased	“[S]ensing, or feeling of spiritual connection” with the deceased.^41^
Visions of a ghost	Frequently described using emic terminology in ethnographic research, such as the term *arutam* for the Jivaro (or Shuar) in the Amazon.^42–44^

Most of the research on SED has occurred within a bereavement context. This has often been separated from research on hallucinations but mirrors the general debate regarding the pathological or non-pathological nature of hallucinations: eg, whether some SED may be associated with psychopathology.^20,21,45,46^ The present review focuses primarily on the literature on spontaneously (ie, involuntarily) occurring SED, and research on induced SED (eg, experiments^47,48^ and drugs^42,43,49^) is considered beyond the scope of this review. 

One of the earliest systematic studies of SED was conducted in the late 19th century as part of the Census on Hallucinations,^50^ and later SED have featured in the works of Freud,^51^ Bowlby,^52^ and Parkes.^53^ Since then, 6 reviews^54–59^ of this field have been published to our knowledge, including 2 summary reviews,^58,59^ 2 reviews focused on the relationship between SED and mental health,^54,55^ a systematic review framing SED as hallucinatory experiences,^56^ and a review of cross-cultural differences in SED.^57^ However, a review that integrates all of these perspectives—encompassing contributions from a range of disciplines—has not yet been conducted, despite the topic clearly warranting this kind of approach.

The present review was prepared by an interdisciplinary working group as part of the International Consortium for Hallucination Research (ICHR). It draws on the different disciplinary backgrounds of its members, comprising psychology, neuroscience, philosophy, and theology, as well as clinical practice. This review seeks to shed further light on the nature of the experiences, including their potential relationship to psychopathology, as well as outlining various theoretical frameworks that should be considered, in working toward a coherent picture that can inform both practice and research. Understanding SED better—and their potential relation to psychopathology—is important for clinical decision-making, normalization of everyday “unusual experiences” in the healthy population, and providing proper care to those whose experiences of the deceased may, in fact, reflect psychosis and psychopathology.

## Phenomenology

SED range from clear and distinct experiences to subtle or partial impressions (see tables 2 and 3 for examples and an overview of prevalence, respectively), which can be placed on a continuum of vividness.^60^^(p160)^ For example, the quasi-sensory feeling of presence is sometimes described as a diffuse “feeling” that the deceased is there and at other times as a clearly locatable sense of presence, as exemplified here: “It was as if he was sitting next to me really.” ^11^ In addition, auditory SED may include both sounds (eg, footsteps) and auditory-verbal experiences (eg, hearing the deceased calling one’s name).^7,37^

**Table 2.** Perceptual Content and Examples of Sensory and Quasi-Sensory Experiences of the Deceased (SED)

Sensory Modality	Perceptual Content	Examples Reported by Perceivers
Sense of presence	The deceased as felt presence that can be located in space	“I just completely relaxed inside this car [.] He was with me. It was as if he was sitting next to me really.” ^11^
	The deceased as nonspecific yet “felt” presence/awareness	“Sometimes I just know he’s around, you know. And other times I don’t. But when I do think that he is it’s such a strong feeling that I’m sure of it...” ^61^
Auditory SED	Hearing the voice of the deceased	“[A]nd I heard my grandma say, ‘it’s at the back, it’s at the back’. And [.] as I looked towards the back I could see there was like a, thing that needed, needed to be turned,” ^3^
	Hearing sounds of the deceased	“I’ve heard odd noises once and once I was frightened. I said, ‘Stop that dad’, and it did stop.” ^37^
Visual SED	Seeing the deceased in full figure	“And, all of a sudden, from nowhere, he appeared! I mean, I just – a vision of him was right in front of me. I mean, it lasted a split second. But, it was there.” ^32^
	Partial visual perception of the deceased	“Well this was an eye and a nostril, it filled the whole of my, my vision bit there, my vision that you can see and like a nostril, and it was all, sort of, floaty and I thought that looked like my mum when she was young.” ^37^
Tactile SED	Feeling touched on specific part of the body	“When I sat alone at the dining table, I felt how she put her arm round my shoulders as she used to do when she served me food.” ^62^
	Feeling held/being enveloped by the deceased	“[A]nd then he gave me this big hug . . . and it was just this intense feeling of peace . . . everything was going to be okay because my grandpa was going to make it okay,” ^29^
	Touching the deceased	“I was reading when suddenly a figure floated over me about a foot above my head. She was wearing a white, long-sleeved night-dress, and her hair looked as brilliant read as it really was. I reached out and stroked her face, which felt just like any other face. The figure at once faded away” ^63^
Olfactory SED	Smells emanating closely from the deceased	“I started to smell cigar smoke, and then out of the corner of my eye I saw someone sitting in the chair. And it scared me, and then I realized it was my grandfather and I felt surprised. Now whenever I travel I smell that cigar smoke and that’s how I know he’s around” ^17^
	Smells more broadly associated with the deceased	“At about 9 p.m., two weeks after Stacy’s death, I was in bed and I started smelling Noxzema; this went on for about two hours. [.] This happened for the entire week. Finally, thinking I was losing my mind, I asked my husband at 9 p.m. one evening if he smelled Noxzema. He said yes, that he thought I had started putting it on like Stacy used to every night before she went to bed.” ^41^
Gustatory SED	Perceived taste of food linked to deceased (in combination with smell)	“And, it was a very strong, powerful smell. Which, I knew wasn’t in the room but I could definitely smell it none the less. Erm, and, erm, sort of a few seconds after that, I could really taste like[.] the food” ^64^

**Table 3.** Overview of Reported Prevalence of Different Types of Sensory and Quasi-Sensory Experiences of the Deceased (SED) at First Available Assessment Point

Prevalence in Relation to the Full Sample								
Reference	Sample	Assessment Method	Sense of Presence	Visual	Auditory	Auditory-verbal^a^	Tactile	Olfactory
Grimby^4,62^	50 spousally bereaved persons	Semi-structured interview	52%	26%	30%	30%	6%	—
Rees^2^	293 spousally bereaved persons	Semi-structured interview	39.2%	14.0%	13.3%	11.6%	2.7%	—
Prevalence in Relation to Subsample Reporting SED								
Reference	Sample	Assessment Method	Sense of Presence	Visual	Auditory	Auditory-verbal^a^	Tactile	Olfactory
Datson and Marwit^1^	87 mixed-bereaved persons	Self-report items	50%	17%	19%	—	10%	4%
Kamp et al^5^	175 spousally bereaved persons	Self-report items	—	52%	45%	32%	—	—
Longman et al^7^	97 mixed-bereaved persons	Open-ended self-report questions	82%	29%	31%	—	10%	10%
Olson et al^8^	46 spousally bereaved persons	Semi-structured interview	32.1%	78.6%	50.0%	17.9%	21.4%	—

^a^Includes hearing the deceased’s voice and/or speaking to/with the deceased.

 This variety mirrors that of “hallucinations” in psychiatric contexts, eg, auditory-verbal hallucinations (AVHs). In the case of AVHs, it is similarly debatable whether and how principled distinctions might be drawn between subtypes.^65^ Variables include volume, degree of personification, level of voice control, and whether the experience is distressing. Voices may take the form of commands, advice, encouragement, comments, and/or abuse.^66–68^ Like AVHs in other situations, hearing voices in bereavement usually involves meaningful language,^3,69^ which may refer to the hearer’s life (past and/or present).^3,70,71^ For example, a bereaved woman may hear her deceased husband complimenting her new haircut.

Hence, comparing SED with similar experiences that arise in psychiatric and other contexts is likely to be informative. For instance, distinctions drawn between subtypes of AVHs have the potential to inform how we interpret and categorize SED and vice versa.^72^ In both cases, there is also a need to clarify what, if anything, the full range of experiences encompassed by the term have in common with one another.^56,72,73^ Furthermore, the task of identifying criteria for distinguishing pathological from healthy forms of experience applies to both, and any proposed criteria should be consistent.

## Who Experiences SED?

SED occur across cultures,^57,59^ in all age groups,^1,2,13,74–76^ and in all types of relationship loss,^3,5,33,41,61,77–79^ regardless of religious affiliation,^1,2,13,59^ and whether the cause of death is natural (eg, disease)^2,5^ or violent (eg, suicide, homicide, and natural disaster).^14,21,80^ In addition, SED may be more prevalent among women^1,4,28,76^ and with increasing age.^2^ However, a curvilinear association between SED and age has also been suggested.^8^ Interestingly, SED among widowed people have been associated with reported pre-death relationship satisfaction and harmony,^2,4^ as well as longer marriages,^2,5^ pointing to a potential impact of the pre-death relationship on the prevalence of SED. However, all of these results are tentative and more research is warranted, as there is a high level of methodological heterogeneity across studies and several of the associations have not been replicated.

Turning to intrapsychic characteristics, SED have been associated with personality constructs, such as openness to experience, neuroticism, and extraversion,^1,2,5^ as well as the tendency to adopt an avoidant coping strategy.^5^ Similar associations have been identified in research on hallucinations,^81^ and on mystical and anomalous experiences.^82^ This points to a potential role for how the individual encounters and views the world, but more research is needed to understand the interplay with such individual differences.

## Welcome, Unwelcome, and Ambivalent Experiences of the Deceased

How SED are anticipated, experienced, and evaluated by the experiencer is interwoven with sociocultural processes.^57^ In some cultures, SED may be feared and regarded as experiences to be avoided, such as among the Navajo in North America^22^ and the Kagwahiv and Matsigenka in the Amazon.^42,44^ In other cultures, SED are even sought after, eg, among Catholic Mexican-American families, where the deceased may be perceived as a guardian angel,^33^ or in a Taoist Hong Kong Chinese sample, where the deceased was sometimes expected to appear in the form of an insect.^15^ However, although sociocultural atmospheres may structure and shape how SED are experienced, they are not exclusively responsible for determining whether the experiences are positive or negative.^57^ For example, a case of a highly distressing SED has been reported from Japan,^46^ a culture offering sociocultural sanctioning such as encouragement of post-death communication and the ritualization of SED around a family altar.^35,83^

Most of the available research on SED, however, has been conducted in a Western context, where a majority of the experiences are considered positive, such as helpful or pleasant.^1,2,5,32,62^ SED may assist the experiencer in solving everyday practical problems, be a source of guidance or encouragement, or ease emotional distress, eg, by helping resolve “unfinished business.” ^3,32^ For some, the experience may also be a source of personal and spiritual growth,^55,60^ as well as serve to reinforce religious worldviews by providing perceived evidence for an afterlife and giving hope for a reunion with the deceased.^11,12,14,60,84,85^ Similarly, experiences outside of bereavement, such as a sense of presence in survival situations,^86^ can involve experienced purpose and may also have a functional role to play.^87^ For example, a presence might be experienced as guiding one to safety during a test of endurance such as a long-distance swim in adverse conditions. The positive quality of many SED seems to differ from, eg, sense of presence during sleep paralysis, which shares with SED the immediacy of the presence in a domestic, everyday environment, with a frequent sense of agency and intention, but often involves malevolent presence, with anonymous identity.^88^

Research conducted in a Western context also points to a significant minority of SED being an ambivalent experience, and to some even negative.^1,2,5,39^ For instance, a positive SED may involve (or be followed by) a painful feeling of absence, which could be intensified when the experience ends or fails to reoccur when wanted.^6,62^ Some SED may be distressing in themselves (eg, seeing the deceased cry or hearing a hostile voice), perceived as intrusive or disturbing in their timing, and/or continuing a difficult (or abusive) relationship.^3,16^ However, distress can also be mediated by how an experiencer responds to SED. Hence, it does not simply reflect the content of the experience, a point that applies equally to certain distressing phenomena in psychosis, such as hostile voices.^3,70,89,90^ Stigma can be a cause of feelings of ambivalence and distress, highlighted by a reluctance to disclose SED among bereaved people in some Western countries.^2,4,16,60^ As such, the experiences may be initially welcome, but worriedness over one’s mental health (due to the perceived association in general society between hallucination and psychosis) as well as other people’s reaction, or the anticipation of it (eg, family and doctors), may cause distress to the individual.^2,16,60,62^

## Multiple Interpretative Resources

Research has also pointed to the relevance of interpretative resources, such as spiritual/religious or psychological frameworks.^11,55,59^ For instance, a Norwegian study identified both rationalist/materialist interpretations (related to secular and some Christian traditions) and supernatural interpretations (related to spiritualist, Buddhist, New Age, some Christian traditions, and folk religious beliefs) as interpretative resources for making sense of SED.^60^ Furthermore, diverse interpretative resources have been identified within the same family^91^ and even the same individual.^31,60,64^ Further research could explore how contextual factors may shape the experience of SED, as well as assimilation and accommodation processes, which may facilitate the integration of SED into individual belief systems.^92,93^In particular, more research directly comparing different cultures in terms of SED and associated factors is needed, as was done in a cross-cultural study comparing British and Japanese mourners, which identified comparable challenges in adapting culture-specific beliefs and practices to the individual situation.^94^

## Psychological Distress and Mental Health Diagnosis

Although SED are often *themselves* sources of comfort, rather than distress,^1,2,56^ research on psychological distress indicates that their presence is associated with higher levels of bereavement-related distress, such as anxiety, depression, and loneliness.^4,5,36,76,95^ More research is needed to understand the interplay and clinical significance of this association, but given the high prevalence and heterogeneous nature of SED, it seems unlikely that SED, in general, serve to indicate psychopathology.^56^

Some attention has been given to the characteristics of SED potentially moderating the distress.^5,39,96^ For example, perceivers reporting either extremely positive or negative SED had more bereavement-related distress compared with experiencers with neutral, to slightly positive appraisals.^39^

### SED and Grief Complications

At this time, 3 labels for grief complications are used predominantly, namely Persistent Complex Bereavement Disorder (PCBD; listed under “condition for further study” in DSM-5),^97^ Prolonged Grief Disorder^98^ (PGD; providing the basis for PGD as included in ICD-11^99,100^ and proposed as part of a revised DSM-5^101,102^), and Complicated Grief (CG).^45,103^ These share the overall characteristics of separation distress and intense emotional pain persisting at least 6 months (eg, ICD-11) or 12 months (ie, DSM-5).^97,100,102,103^

Two studies exploring the diagnostic symptoms of CG found visual and auditory-verbal SED to be a poor *identifier* of CG, but did find that people with these experiences tended to display very intense levels of grief.^45,104^ The suggestion that visual and auditory-verbal SED may be indicative of a more severe type of CG,^45^ resulted in their appearing in DSM-5 under “associated features supporting diagnosis” of PCBD.^97^ Subsequently, a positive association between frequency of visual and auditory SED and symptom level of PCBD was reported.^105^ Most recently, experiencers of SED have been reported to have higher levels of prolonged grief symptoms compared to non-experiencers 4 years post loss.^5^ Notably, the clinical significance of the association was not assessed in the latter 2 studies, and SED are not mentioned in ICD-11^99^ nor in the proposed revision of DSM-5 in relation to the PGD diagnosis set to replace PCBD.^101,102^ More research is needed to verify the suggestion that some SED may be associated with severe grief complications, using validated measures of SED and employing a design that is sensitive to more specific characteristics of SED that may be diagnostically relevant, such as modality, frequency, persistence, and level of distress (in themselves or in combination).

### SED in Relation to Psychosis

Relations between SED and psychosis, including schizophrenia, are poorly understood. To our knowledge, no empirical studies to date have examined them directly. A psychotic variant of PCBD has been suggested where a severely abusive, persistent, and distressing auditory-verbal expression of SED was identified as a key feature,^46^ but this is only based on 1 case study. Given this lack of knowledge, future studies are clearly needed that examine this important issue. For instance, what is the prevalence and nature of SED in individuals with a psychosis diagnosis, and do they indicate anything clinically significant in this population (eg, in terms of prognosis, severity of symptoms, and treatment)? In addition, future studies might explore how people with a psychosis diagnosis who experience SED manage their experiences compared with individuals with other diagnoses and those in nonclinical populations. Finally, for early detection: does the presence of SED in prodromal phases contribute in any way to increasing the likelihood of developing psychosis?

One important difference between voices in bereavement and in psychosis is that the former are seldom anonymous and are instead clearly linked to the hearer’s biography and past relationships, as when someone hears her deceased grandmother’s voice soothing her to sleep just as she did in her childhood. By contrast, in cases of non-bereavement voices, connections to persons or events in the voice-hearer’s life are often less obvious, indirect, or symbolic.^3,106^ For example, a person diagnosed with psychosis might hear an anonymous voice apparently out of nowhere, but on closer inspection, the voice recreates verbal interactions with a critical parent or school bully. In contrast, the quality of a recognizable identity in SED is shared with presences during non-life-threatening solo pursuits (eg, long-distance running, caving, or diving), such as feeling as if a relative is in the cave with you.^87^

A further question concerns *reality testing*. Hallucinations in schizophrenia have been linked to impaired reality monitoring (ie, *the ability* to distinguish an event as externally perceived or internally generated), based on source-monitoring experiments.^107^ However, to our knowledge, no such research has been conducted among experiencers of SED. A related definition of reality testing, concerning whether *a perception* is recognized as “true” or “false,” sometimes marked by the distinction between *hallucination* and *pseudohallucination*, has been discussed in relation to SED, where impaired reality testing has been observed in a few cases.^19^ However, it is questionable whether this distinction is sufficiently sensitive to the full diversity of SED, particularly in light of the impact of culture and belief on the interpretation of these experiences (eg, belief in an afterlife).^11,60^ Similar concerns could be raised about hallucinations in psychosis. For example, “voices” outside of bereavement are sometimes experienced as occurring in a subtly different reality that overlaps with consensus reality; they are “real,” but not in quite the same *way* as the voice of someone next to the hearer.^72,108^ Hence, more nuanced distinctions may be needed to accommodate the complexity and heterogeneity of both phenomena^64,70^. Research using the concept of “mundane” reality testing^70^ (eg, everyday actions someone might perform in response to a voice, such as checking if someone is standing behind them) suggests preserved reality testing in SED, in the sense that the experiencer knows the deceased has died, and that others may not share their experiences of the deceased.^60,64^ Future research may explore this question using a variety of methodologies, eg, testing continuum models of psychosis.^109^

## Theoretical Perspectives on SED

Besides the suggestions that some experiences of SED may be indicative of disordered grief, another 4 central theories are presented in the literature to account for or explain SED, most of which take a relational perspective on the experiences. However, it should be noted that theories of SED are aimed at offering different things, such as establishing causality, understanding why and when SED are more likely to occur, or offering frameworks for individuals to make the content of their experiences intelligible and meaningful.

### SED as Intrusive Symptoms

SED have not explicitly been described as trauma symptoms, but a comparison to post-traumatic stress disorder-like intrusion has been made.^21^Within this theory, very vivid visual, auditory-verbal, and tactile SED are treated as a sign of the bereaved person failing to fully integrate their experience of loss.^21^ This is reflected in the design of questionnaire items that ask about very literal experiences, such as “I actually saw the deceased stand before me.” However, this perspective has limited utility to account for SED, as it overlooks the variety of SED, as well as the manner in which SED are reported by many experiencers, which tends to be tentative as to the “location” of the experience within imagination or shared reality. Qualitative research shows that experiencers often use language such as “it was *as if* he was sitting next to me really”(emphases added).^11^ Further, although some experiences have distressing qualities, most of the SED do not indicate intrusiveness. Lastly, in contrast to “flashbacks,” SED often contain new features and arise in new circumstances and tend to evolve with the experiencer, often reflecting the time and changing circumstances since the death.^3^

### SED as Attachment-Related Responses

Within an attachment perspective, SED have been theorized to arise during the desperate seeking that follows the loss of an important attachment figure.^52,53,110^ The deceased, who is absent and longed for, is found, albeit briefly. This theory accounts for the seemingly higher prevalence in both “closer” relationships^2,79^ and the first few months of bereavement.^4,39^ However, the theory does not account for SED occurring later,^11,60,64^ sometimes years after the death, when experiencers are not clearly in a “seeking” mode of consciousness (or unconsciousness). Future research may assess the significance of the loss, the nature of the attachment to the deceased, as well as time since the loss at the first occurrence of SED.

### SED as Continuing Bonds

Some authors have suggested SED as particularly vivid expressions of a continuing bond with the deceased.^32,111^ The continuing bonds perspective, which arose in response to 20th-century Western assumptions that grief required the gradual relinquishment of ties to the deceased, suggests that maintaining one’s connections with the deceased is normal and can be of benefit to the bereaved.^112^ Different forms of SED are conceived of as reflections, and also continuations, of certain aspects of the pre-death relationship with the deceased, which are shaped by different cultural contexts.^11^ It also allows for a variety of consequences of SED, from the soothing presences that reflect broadly supportive relationships, to the unwelcome presences that continue the aspects of hostile relationships.^3^ It thus provides a framework that links the diversity of SED with the diversity in the biographical, relational, and sociocultural contexts of the experiencers’ lives. However, it does not seek to explain people’s experiences in terms of individual differences and intrapsychic propensities.^83^ Future research may explore relationship features continuing or being transformed through SED.

### SED as Part of a Dialogical Self

Based on Dialogical Self Theory,^113,114^ SED are suggested to appear in the external domain of the self.^60^^(p267)^ In contrast to the internal domain, which consists of “internal I-positions,” the external domain is populated by “the other in the self” constituting a “society of mind” ^114^^(p2)^ providing a space for both real and imaginal others.^114^^(p18)^ Dialogical Self Theory holds that there are porous lines between internal and external domains, as well as between the extended self and outside domain.^115^ This porosity may account for common reports of bereaved individuals experiencing their deceased in an imaginal-perceptual space between the inside and outside worlds.^11,37,60^ Further, as the dialogical self is pictured as multi-voiced and consisting of decentralized I positions,^114^^(p3)^ it gives theoretical underpinnings to understand how bereaved individuals can experience their deceased as dead—yet from another position, they experience them as alive.^11,37,60^

### SED as Survival of Consciousness

Although controversial in mainstream research, there is a long tradition of sampling and studying SED from a parapsychological perspective, being open to the possibility of survival of consciousness as a possible explanation of SED.^38,50^ Here, case reports, such as multiple witnesses of SED or the bereaved receiving veridical information from the deceased, have been examined. However, it is debatable what would constitute empirical evidence for “survival,” satisfying criteria for objective verifiability, and whether such evidence is attainable.^85^

## Clinical Implications

Based on the current state of knowledge as summarized in this review, including case studies^116–118^ and reports from therapists and clients^16,119^ regarding what has been found to be helpful or unhelpful,^16,26,120,121^ clinical recommendations have been compiled in table 4. Future research should include systematically conducted outcome studies for working with distressing SED as well as mixed-method process research to identify key features of best practice.

**Table 4.** Clinical Recommendations for Assessing and Working With Sensory and Quasi-Sensory Experiences of the Deceased (SED)

1.Assessment, Diagnosis, and Risk	
1.1. Assessment of SED	When SED have been disclosed, clinicians should: - be welcoming of clients disclosing SED - allow detailed narrative retelling of the event(s) - assess for the impact of the experience on the client - explore the meaning of SED to the client
1.2. (Preexisting) mental health problems	In cases of (preexisting) mental health problems, or clinical deterioration, clinicians should: - assess (prior) mental health problems and possible links to current context - be cautious as in some cases distressing SED may be linked to preexisting mental health issues or clinical deterioration - be aware of the risk of misdiagnosis, given that the vast majority of SED tend to be benign
2.Therapeutic Strategies	
2.1. Psychoeducation	If bereaved disclose concerns for their sanity due to SED, clinicians are advised to: - normalize SED by sharing information, eg, the high prevalence of SED - provide reassurance that SED are not normally linked to mental health problems
2.2. Relationship reprocessing	Working with the relationship, eg, in grief therapy: - SED can be used as a catalyst for working on the relationship with the deceased - SED can lead to developing a helpful continuing bond, although not necessarily in all cases
2.3 Working with welcome SED	When SED are welcome: - SED can be used for drawing on the continuing bond with the deceased as a resource for coping with grief - meanings and messages can be taken forward into the client’s ongoing life
2.4. Working with unwelcome or ambivalent SED	In cases of unwelcome or ambivalent SED, practitioners should: - carefully assess the circumstances of SED - assess the current context of the client - assess the context of the pre-death relationship - explore potential to work on unfinished business with the deceased person - offer opportunities for exploring different ways of responding to SED
2.5. Addressing existential crisis/cognitive dissonance	Practitioners should be willing to explore issues such as: - struggle to make sense of SED - lack of available conceptual frameworks within which the experience can be understood - clashes between experience of SED and beliefs including spiritual and religious beliefs
3. General Guidelines for Clinicians	
3.1. Nonjudgmental exploration	Clinicians should approach clients’ experiences: - with openness - in a nonjudgmental manner - with respect for the client’s perception and interpretation
3.2. Cultural sensitivity	Clinicians should: - pay careful attention to how clients make sense of the experience - pay attention to the language clients use to frame their experiences - respect the worldview of the client - be sensitive toward clients’ social and cultural context - be open to diverse perspectives
3.3. Affirmative stance (if relevant)	If appropriate and relevant, clinicians should: - adopt an affirmative stance towards SED - help clients find the transformative potential in SED - reinforce posttraumatic growth if naturally occurring in client’s response to SED

## Synthesis

The present review offers an interdisciplinary perspective, drawing on current evidence and theoretical models of SED as presented across diverse subfields of relevant research, including psychotherapy, clinical, social and counseling psychology, psychiatry, parapsychology, anthropology, and psychology of religion and philosophy.

SED have been associated with bereavement-related distress, but more research is needed here, and the evidence and associated critiques presented in the current review point to SED as a common and normal occurrence in bereavement, comforting or reassuring to most, the majority of the time. Importantly, the meanings and consequences of SED should be viewed with reference to the individual, their relations to the deceased, and their cultural context (see table 4 for clinical recommendations).

Suggestions for future research have been provided throughout this manuscript. We encourage researchers to follow up on these and other ideas in rigorous future studies. Furthermore, as SED are wide-ranging experiences, this raises a number of challenges for clinicians and researchers seeking to assess their occurrence. In light of this, some of the main methodological limitations prevalent in SED research should be highlighted: First, there is considerable heterogeneity when defining SED as a phenomenon, reflected in the many terms used (see table 1). Second, there is currently no validated measure of SED, with different items and specifications being used across studies (see table 5). Third, recruitment methods often consist of advertisements in relevant subpopulations, which may result in biased samples with extreme experiences on various parameters. In this context, more research with representative samples of the general population (ie, epidemiological studies) is needed in order to avoid such a sample bias.

**Table 5.** Assessing Sensory and Quasi-Sensory Experiences of the Deceased (SED) Using (A) Self-Report and (B) Interviews

A. Self-report	Example Questions	Response Format	Sample
Byrne and Raphael^122^	“Have you felt as though you have seen her, heard her, or felt as though she has touched you?” and “Have you felt as though she is still present?”	Four-point frequency scale ranging from “never” (0) to “often” (3) in respect to the previous 2 weeks.	78 spousally bereaved men
Datson and Marwit^1^	“It is not uncommon for bereaved people to report sensing the presence of a deceased loved one. The following questions relate to this experience. Please answer as honestly as possible by checking the appropriate response. Thank you. In the time since the death of your loved one, have you ever felt a sense of their presence?” “In what way did you sense your loved one’s presence?”	Yes No *Select from:* “Sight,” “smell,” “sound,” “touch,” “by general awareness only, without a specific cause.”	87 mixed-bereaved persons
Epstein et al^34^	“I sometimes ‘see’ him even though he is dead”, “I sometimes ‘hear’ him even though he is dead” and “I sometimes feel his presence even though he is dead”	The response format was not specified, but the items are based on the Bereavement Experience Index (BEI),34 which has a 6-point “true/false-scale.”^123^ The present items were not included in the published version of BEI.^123^	45 spousally bereaved persons
Houck^13^	“After the death of your loved one, was there ever a time when you sensed his/her presence?”	*Select from:* “Sense of the loved one being in the same room,” “Olfactory sensation, such as familiar scents, perfumes, or odors,” “Auditory sensations, such as voices, footsteps, or music,” “Visual sensations, such as seeing an outline or shape,” “Tactile sensation, such as feeling a presence through touch.”	162 mixed-bereaved persons
Jahn and Spencer-Thomas^14^	“Did you have any ‘spiritual experiences’ with the person you lost to suicide after the death?” “What was the form of spiritual contact you experienced (select all that apply)?”	Yes No *Select from:* “Feeling the presence of the person,” “Seeing a vision/image of person while awake,” “Hearing person’s voice while awake,” “Smells related to person” (other types of contact was also listed, eg, “unusual animal/insect behavior”).	1301 mixed-bereaved persons
Larøi and Van der Linden^124^	“On certain occasions I have had the feeling of the presence of someone close who has deceased” Items from an extended version of the Launay-Slade Hallucinations Scale	Five-point Likert response scale: 0 =“certainly does not apply to me,” 1 = “possibly does not apply to me,” 2 = “unsure,” 3 = “possibly applies to me,” and 4 = “certainly applies to me.”	236 non-bereaved persons
Lee^105^	“Temporarily thought that you saw or heard the deceased”	Five-point frequency scale from “not at all” (0) to “nearly every day” (4).	135 mixed-bereaved persons 228 mixed bereaved persons
Longman et al^7^	Open-ended self-report item: “People sometimes feel that they sense the presence of their loved ones after death. These experiences can occur in several ways. Hearing, seeing, smelling, touching, or a special sense of nearness. What happened?”	Open ended response.	97 mixed-bereaved persons
Field and Filanosky^21^	“I actually heard the voice of the deceased speak to me”, “I actually felt the deceased’s physical touch” and “I actually saw the deceased stand before me”	Four-point frequency scale ranging from 0 to 3, with respect to the previous month.	502 mixed-bereaved persons
Simon et al^45^	“I see the person who died stand before me” “I hear the voice of the person who died speak to me”	Five-point frequency scale ranging from “never” (0) to “always” (4) with respect to previous months.	782 mixed-bereaved persons
Bennett and Bennett^31^	Interview study: Questions about the “presence of the dead” were asked when the context seemed to allow it. Questions were couched in vague and neutral terms, such as “Do you ever feel he’s still around?” “Do you ever feel his presence?” and “Do you ever feel the presence of your husband?”	Descriptive, categorizing narratives by sense modality, and discourse analysis.	19 spousally bereaved women
Chan et al^15^	Client-centered counselors conducted semi-structured in-depth interviews focused on continuing bonds and bereavement as part of the therapeutic process.	Consensual and group-based thematic analysis.	52 mixed-bereaved persons
Conant^32^	Questions about “experiences of remembering” and “feelings and imagery” of their deceased husbands, taking a closer look at their sense-of-presence experiences.	Narrative analysis including development of composite vignette and heuristic models for the role of sense of presence in grief.	10 spousally bereaved women
Doran and Downing Hansen^33^	Ethnographic fieldwork and semi-structured interview focusing on the family’s grief and meaning-making after losing a family member. They also used drawings when interviewing children.	Thematic analysis.	9 mixed-bereaved people
Gondar-Portasany^125^	Ethnographic fieldwork and biographical interviewing conducted over several years. People were asked about “apparitions of the deceased.”	Ethnography.	1873 mixed-bereaved people
Grimby^4,62^	“Have you ever felt that your husband/wife has been with you in some way since he/she died?” If confirmed: “In what way?”	Interviewer rating of illusions (ie, the deceased are present in the room) and hallucinations (ie, visual, auditory, tactile, and conversations with the deceased).	50 spousally bereaved persons
Hayes and Leudar^3^	Participants were told that the interviewer was interested in experiences of continued presence in bereavement and would like to hear about what had happened to them.	Ethnomethodological conversation analysis.	17 mixed-bereaved persons
Klugman^28^	“Do you have a connection with someone who has died?,” “Do you ever suddenly notice the smell of a deceased person’s smell, and associate it with the deceased?,” “Do you ever suddenly hear their footsteps that you associate with the deceased?,” “Do you ever suddenly hear their voice that you associate with the deceased?,” “Do you ever feel the deceased’s touch?,” “Do you ever have a vision or image of the deceased?,” “Do you ever feel the presence of the deceased?” and “Do you ever converse with them?”	Interviewer registering of response.	202 bereaved and non-bereaved persons
Keen et al^37^	Interview questions included: “How would you describe what having this experience means to you?” Prompt: “What did you believe was happening? How do you make sense of what was happening? Do you have any explanations about these experiences?”	Interpretative phenomenological analysis.	8 mixed-bereaved women
Nowatzki and Kalischuk^29^	Interview questions asked participants to describe their encounter(s) with the dead, what meaning the experience had for them, and impact on grieving and beliefs.	Hermeneutic-phenomenological analysis.	23 mixed-bereaved persons
Olson et al^8^	“Have you ever experienced your husband/wife with you in any way since his/her death” and “Have you ever had other such experiences?”	Interviewer rating of illusion and hallucinations (ie, visual, auditory, talking with, and tactile).	52 spousally bereaved persons
Parker^17^	Questions focused on the lived experience of having had an “extraordinary experience” in bereavement as well as experiencers’ beliefs, effects, and usages of the experience.	Content analysis, leading to the development of a cause and effect network.	12 mixed-bereaved persons
Steffen and Coyle^11^	Participants were asked to describe their experiences and what they meant to them as freely as possible while exploring a range of potentially significant dimensions as suggested by relevant theory and research; eg, how presence-sensing might impact on the survivors’ relationships with the deceased and others, their sense-making regarding the death, their belief systems, and sense of self.	Thematic analysis.	12 mixed-bereaved persons
Troyer^30^	Core question asked: “Have you ever seen a vision of your wife, heard her talking to you, or experienced a touch or smell that made you believe that she was nearby?”	Naturalistic inquiry.	6 spousally bereaved men
